# Supplementary material for: Polyamine Homeostasis in Snyder-Robinson Syndrome
Source: Med Sci (Basel). 2018 Dec 7;6(4):112. doi: 10.3390/medsci6040112 (PMC6318755; doi:10.3390/medsci6040112)
Supplement: Supplementary file 1 [file medsci-06-00112-s001.pdf]

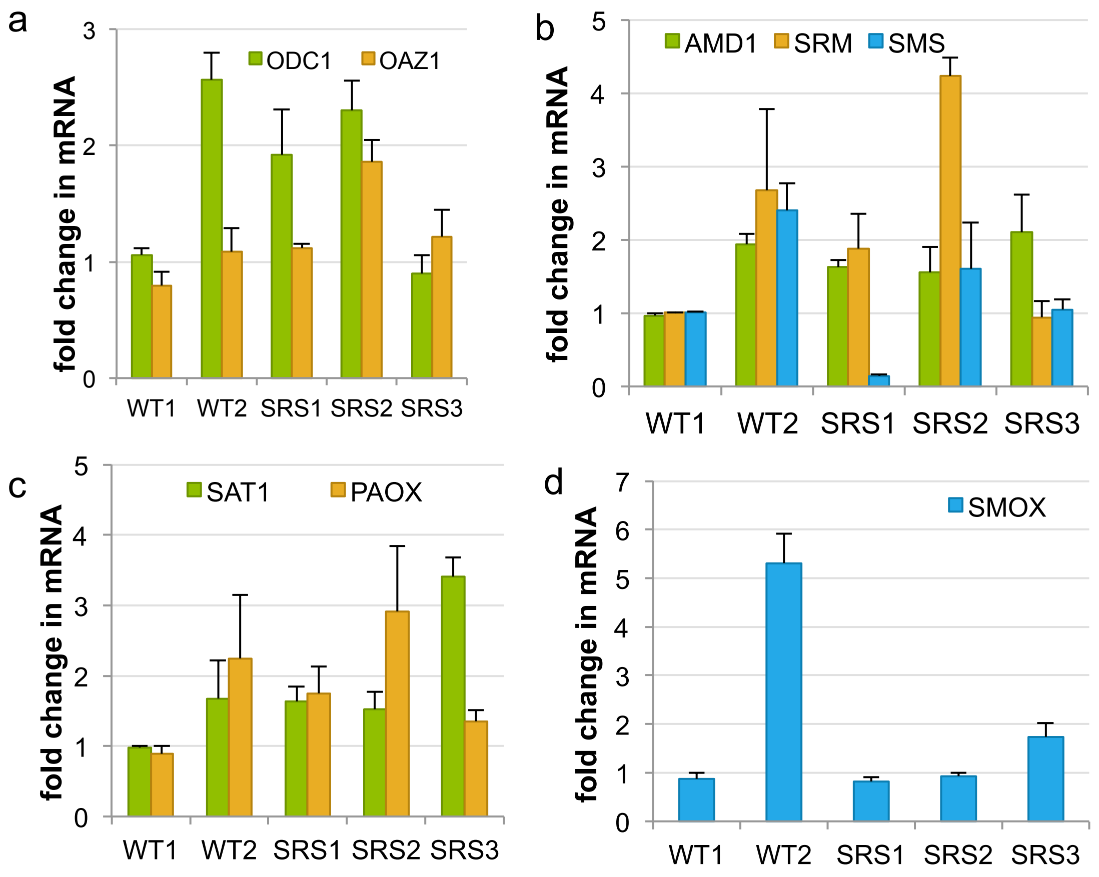

Figure S1. Quantitative RT-PCR results for (a) *ODC1* and *OAZ1,* (b) AMD1, SRM, and SMS, (c) *SAT1* and *PAOX*, and (d) *SMOX* mRNA expression, normalized to *GAPDH* (n = 3; error bars = SEM).
